# Alcohol use disorder and co-occurring mental illness among older adults in low-and middle-income countries: a narrative systematic review

**DOI:** 10.1186/s13722-025-00640-4

**Published:** 2025-12-27

**Authors:** Nebiyu Mengistu, Carmen Axisa, Priya Saravanakumar

**Affiliations:** 1 School of Nursing and Midwifery, Faculty of Health, University of Technology Sydney, Ultimo, New South Wales Australia; 2 Department of Psychiatry, College of Medicine and Health Sciences, Dilla University, Dilla, Ethiopia

**Keywords:** Older adults, Alcohol use disorder, Co-occurring mental illness, LMICs

## Abstract

**Background:**

Alcohol use disorder (AUD) and co-occurring mental illness present complex and growing challenges among older adults, particularly in low-and middle-income countries (LMICs), where health systems often lack adequate resources and tailored interventions. Despite the rising prevalence of dual diagnosis in older adults, research on this issue remains limited in LMICs contexts.

**Method:**

A narrative review with systematic search was conducted and the reporting was adapted from the preferred reporting items of 2020 for systematic reviews guidelines where applicable. Literature was searched using online databases including PsycINFO, MEDLINE(Ovid), Embase (OVID), CINAHL, Scopus, and Web of Science. A google scholar search engine and reference lists of relevant studies were also manually searched.

**Result and discussion:**

The included studies reported substantial variation in prevalence rates of alcohol use and dual diagnosis among older adults, reflecting differences in definitions, measurement tools, and population characteristics. Gender differences were consistently identified, with men more likely to engage in alcohol use, while women experienced higher rates of depression and cognitive impairment. Key associated factors included lower education, living alone, chronic illness, tobacco use, and psychosocial stressors. Mental health outcomes commonly co-occurring with AUD included depression, cognitive impairment, poor sleep and suicidal ideation. Significant gaps exist in LMIC research on the care needs of older adults and healthcare providers in managing dual diagnosis. Most existing studies are cross-sectional and rely on self-reported data, with limited attention to culturally responsive interventions.

**Conclusion:**

This review shows that AUD and co-occurring mental illness are significant yet understudied public health challenges. It highlights gender differences in drinking patterns, multiple psychosocial and health related contributors in LMICs. Most available studies are cross-sectional and concentrated in a few settings, which limits broader interpretation. Future research should focus on more rigorous and culturally grounded research is needed to better understand these complexities and guide appropriate interventions.

## Introduction

Older adults often defined as 60 or 65 years and above for statistical and administrative purposes [1]. However, the classification can sometimes vary depending on the context and specific regional considerations [2]. In many countries, the age of 60 or 65 years also marks retirement, which often brings about significant life transitions. This stage of life is significantly associated with a range of social problems, including social isolation, economic dependency, reduced social status and limited access to essential services [3].

The global population is rapidly aging. In 2020, there were 1 billion people aged 60 years older worldwide [4]. Approximately 14% of adults aged 60 and over live with mental illness [5]. The relationship between mental illness and alcohol use is complex [6].

Alcohol use disorder (AUD) is defined as a problematic pattern of alcohol consumption that results in clinically significant impairment or psychosocial distress within 12-month period [7]. The co-occurrence of AUD and mental illness presents unique challenges in health care, particularly among older adults. These co-occurring mental illnesses can have a bidirectional relationship, mental illness may make a person more likely to use alcohol as an attempt to self-medicate symptoms associated with their mental illness [8]. Conversely, alcohol use may be a contributing factor for the initial symptoms of mental illness [9].

In this population, depression and cognitive impairment are the most frequently identified co-occurring mental disorders. These conditions related to AUD in older adults are often underrecognized [10]. Comorbidities have significant psychological, social, and economic consequences. This places a double burden on older adults, who care for them, and the community [11], yet early detection has the potential to improve intervention [12].

Research undertaken in India showed that AUD was also more prevalent among rural residents (7.9%) than their urban counterparts(6.7%) [13]. A study done in Nigeria indicated that the prevalence of lifetime alcohol use was 69.8% and the current alcohol use was 45.5% [14]. Research done in Ethiopia revealed that the magnitude of AUD among older adults was 27.5%-36.2% [15–17].

Alcohol use disorder and co-occurring mental illness present significant challenges to mental health services. The presence of both conditions are associated with poor prognosis, high rates of psychiatric relapse, medical comorbidity, higher rates of active suicidal behaviours and social isolation relative to individuals with either disorder [18]. Although these risks exist, older adults in low-and- middle income countries (LMICs) are less likely than younger adults to be screened, assessed and managed for AUD and co-occurring mental illness [19].

A large body of literature shows the magnitude, impact and evidence based effective treatment options for co-occurring disorders among younger individuals [20, 21]. However, the rapidly increasing number of older adults has received little attention when it comes to describing the burden of AUD and co-occurring mental illness and treatment options among older adults in LMICs. Studying AUD and co-occurring mental illness among older adults is crucial to providing information required to improve health outcomes of AUD and co-occurring mental illness for future research. Therefore, this review aimed to synthesize existing literature on AUD and co-occurring mental illness among older adults in LMICs with a focus on identifying key patterns, gaps and implications related to prevalence and patterns trends, associated risk factors and psychosocial or mental health outcomes.

## Methodology

## Study design and approach

A narrative review with systematic approach was conducted. This approach was selected because the included studies were highly heterogeneous in outcome measures, definitions and assessment tools. The review was reported with the references to the PRISMA 2020 guidelines where applicable [22]. The review protocol was registered on PROSPERO (CRD420251046004).

## Eligibility criteria

The review considered all original studies conducted on AUD and co-occurring mental illness among older adults to be eligible, where both conditions (dual diagnosis) were addressed. The full inclusion and exclusion criteria were presented in (Table 1).

**Table 1:** Inclusion and exclusion criteria of studies

Criteria	Inclusion criteria	Exclusion criteria
Population	Studies conducted among older adults (aged 60 years and above) in LMICs	Studies primarily focused on younger and middle-aged populations (under 60 years of age)
Exposure	Studies involving older adults with dual diagnosis (AUD and co-occurring mental illnesses)	Studies focused on older adults with SUD other than alcohol use disorders
	Studies where ≥ 50% of the sample has alcohol use as the primary diagnosis, even if there are comorbidities with other substances	Studies where AUD is not the primary condition (e.g., mixed substance use)
Outcome	Any Studies reporting prevalence and associated risk factors, dual diagnosis interventions, perspectives of the management, any barriers and facilitators to managing AUD and co-occurring mental illnesses	
Study type	Peer-reviewed original quantitative, qualitative studies and mixed type of studies	Reviews, case studies, Book chapters, Conference abstracts Study Protocols, unpublished studies including theses, dissertation and grey literature.
	Written in English language	Written in other languages than English
		Research studies that are not downloadable/accessible from the authors.

## Information sources and search strategy

A structured search was developed with support from a university librarian, using targeted key words and phrases within titles and abstracts. Searches were conducted across major databases, including Medline (OVID), Embase (OVID), Web of Science, PsycINFO, CINAHL and Scopus on 21 December 2024. Each database was searched systematically using Boolean operators and algorithms.

For studies not captured through data base searches, additional manual searching was undertaken using google scholar, supported by snowballing to identify related publications. References lists of all eligible studies from both database and manual searches were also screened to ensure no relevant literature was missed. The detail of search strategy is found in (Table 2).

**Table 2:** Keywords/Search terms used in this review

PCC	Keywords/search terms	
Concept 1 (Population)	(“Older adult*” OR “Older individual*” OR aged* OR “aging population” OR aging OR “Aged, 80 and over” OR senior* OR geriatric* OR “older people*” OR “older persons” OR elder*)	
Concept 2 (Exposure):	(Alcohol* OR Alcoholism OR “Alcohol drinking” OR “Alcohol addiction” OR “Alcohol abuse” OR “Alcohol consumption*” OR “Alcohol use*” OR “Alcohol misuse” OR “drinking behav*” OR “Alcohol-related disorder*”)	AND (“Mental illness” OR “Mental disorder*” OR “Mental disease” OR “Dual diagnosi*” OR “Dually diagnosi*” OR Psychiatr* OR Depressi* OR Anxiety OR Bipolar OR Psychotic* OR Psychoses OR Schizophreni* OR “Mood disorder*” OR “posttraumatic stress disorder” OR PTSD or “Psychiatric patient*” or “Mental health”)
Concept 3 (Settings)	(afghan* OR africa* OR albania* OR algeria* OR angola* OR antigua* OR barbuda* OR argentin* OR armenia* OR aruba* OR azerbaijan* OR bahrain* OR bangladesh* OR bengal* OR bangal* OR barbados* OR belarus* OR benin* OR bhutan* OR bolivia* OR bosnia* OR herzegovin* OR botswan* OR brazil* OR brasil* OR bulgaria* OR burkina* OR burundi* OR “cabo verde*” OR “cape verde*” OR cambodia* OR cameroon* OR chad* OR chile* OR china* OR chinese OR colombia* OR comoro* OR comore* OR congo* OR zaire* OR “costa rica*” OR “cote d’ivoir*” OR “cote divoir*” OR “ivory coast*” OR ivorian* OR croatia* OR cuba OR cuban OR cubans OR “cuba’s” OR cyprus* OR cypriot* OR czech* OR djibouti* OR dominica* OR ecuador* OR egypt* OR “el salvador*” OR salvadoran* OR guinea* OR eritrea* OR estonia* OR eswatini* OR swaziland* OR swazi* OR ethiopia* OR fiji* OR gabon* OR gambia* OR ghana* OR gibraltar* OR greece* OR greek* OR guatemala* OR guyana* OR haiti* OR hondura* OR hungary* OR india* OR indonesia* OR iran* OR iraq* OR jamaica* OR jordan* OR kazakh* OR kenya* OR “korea*” OR kosovo* OR latvia* OR lebanon* OR lebanese* OR liberia* OR libya* OR lithuania* OR macedonia* OR madagasca* OR malawi* OR mali OR mauritania* OR mexico* OR montenegr* OR morocco* OR mozambique* OR namibia* OR nepal* OR “new caledonia*” OR niger* OR oman OR pakistan* OR palestin* OR paraguay* OR peru OR philippine* OR poland* OR portugal* OR “puerto ric*” OR romania* OR russia* OR soviet* OR rwanda* OR samoa* OR “saudi arabia*” OR saudi OR senegal* OR serbia* OR “sierra leone*” OR slovak* OR sloven* OR melanesia* OR somali* OR “sri lanka*” OR sudan* OR syria* OR tajik* OR tanzania* OR togo OR tunisia* OR turkiy* OR uganda* OR ukrain* OR uruguay* OR uzbek* OR venezuela* OR vietnam* OR zambia* OR zimbabwe* OR rhodesia* OR “arab* countr*” OR “middle east*” OR “global south” OR sahara* OR subsahara* OR “west indies*” OR caribbean* OR “central america*” OR “latin america*” OR “south america*” OR “central asia*” OR “north asia*” OR “northern asia*” OR “southeastern asia*” OR “western asia*” OR “east europe*” OR “developing countr*” OR “developing nation*” OR “developing world” OR “middle income countr*” OR “low income countr*” OR lmic OR lmics)	

The detail of search strategy is provided in (Table 2).

## Data collection process

After downloading all search results to the desktop, the number of outputs was imported to the review platform Covidence, which automatically removed duplicate records before screening. Two reviewers (N.M. and C.A.) screened each record independently using Covidence review management software. Initial screening composed of reading titles and abstracts of each record. Any conflicts were resolved through consultation with a third reviewer (P.S.). Thereafter, two reviewers (N.M. and C.A.) independently screened full-text articles, with any discrepancies were resolved through consultation with a third reviewer (P.S.).

## Data extraction process

A Microsoft excel spreadsheet was developed to collect relevant information from the selected studies. Two reviewers independently extracted data, resolving disagreements by consultation with a third reviewer (P.S.). The outcomes were iteratively identified during data extraction, since it was unclear beforehand which outcomes had been studied.

Data were extracted on the prevalence and patterns of AUD and co-occurring mental illness, types of mental illness co-occurring with AUD, level/severity of AUD, sociodemographic and psychosocial factors. Information on assessment and management practices was sought but not reported in the included studies.

Key study characteristics were also extracted including author, year of publication, study aims, country study settings (facility based or community based), study type/source, sample size, inclusion and exclusion criteria, data collection procedures, methods of data analysis. Additionally, information extracted including screening tools, independent variables, study impact, key findings, limitations and recommendations.

## Data analyses

The synthesis process was conducted as a narrative Synthesis Without Meta-analysis (SWiM) approach was employed, with reporting guided by the PRISMA guidelines where applicable [22]. This approach was selected because there was insufficient data and marked variation in the definition, measurement tools and outcome indicators used to assess both AUD and co-occurring mental illness. Studies included a broad range of screening tools (e.g. CAGE, AUDIT, ASSIST and Michigan alcohol screening tools) clinical diagnostic tools like DSM, ICD-10) and self-reported non standardized measurements. These screening differences resulted in diverse classification of both alcohol related and mental health outcomes, such as lifetime alcohol use, current use, heavy drinking, alcohol abuse or dependence studied with different mental health outcomes.

Given these variations, narrative review with systematic search allowed for comprehensive exploration of the research findings, identifying common patterns, contextual influences and gaps in the current evidence base across LMICs. The results were grouped by outcome themes, and the certainty of evidence was not graded through a framework, since it is difficult to do so when vote counting. Studies were thematically grouped through an inductive process based on conceptual similarity and recurring patterns. Study characterises and key findings were summarized in tables and figures to support narrative synthesis.

The methodological quality and risk of bias of the included studies were evaluated using Critical Appraisal Skills Programme (CASP) checklist appropriate for observational study design and it was independently reviewed [23].

## Result

## Study selection

**Fig. 1:**
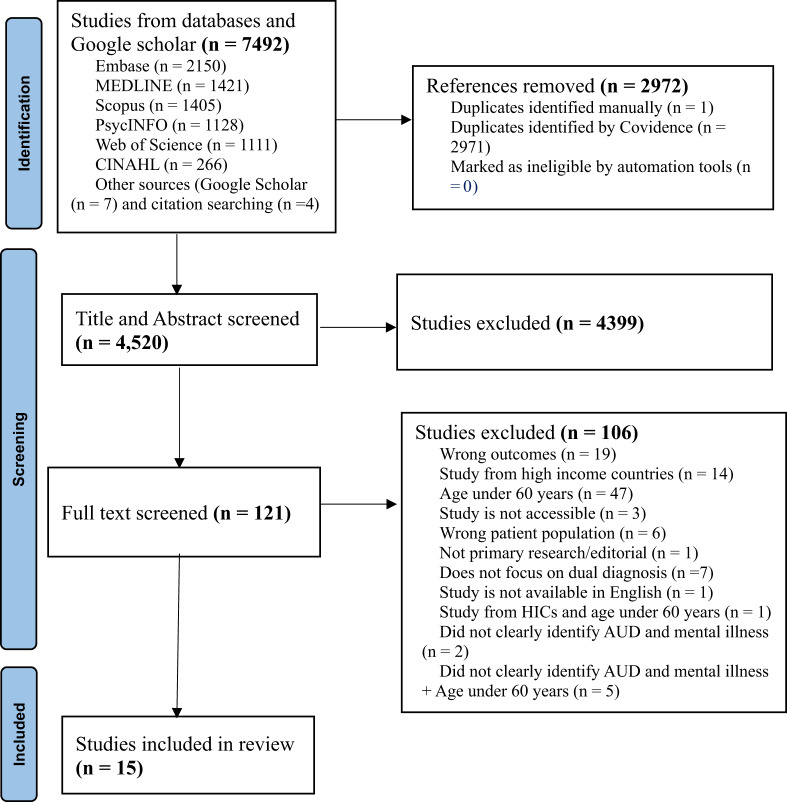
PRISMA flowchart showing the selection of studies

Through a search of selective academic databases, 7492 studies were identified. After removing 2972 duplicates, 4520 articles were retrieved for title and abstract screening. During this process, 4399 articles were excluded, leaving 121 articles for full-text screening. In the full text screening phase, 106 articles were excluded for not meeting the inclusion criteria, resulting in 15 articles being included in this study (Fig. 1).

## Identification of studies via data base search

### Study characteristics

A detailed summary of the study characteristics is provided in Table 6. We included a total of fifteen [15] observational studies investigating in the areas of alcohol use disorders and co-occurring mental illness among older adults. Of these, twelve [12] were cross-sectional studies [13, 15, 24–26, 28–32, 34, 35] and three [3] were cohort studies [27, 33, 36].

**Fig. 2:**
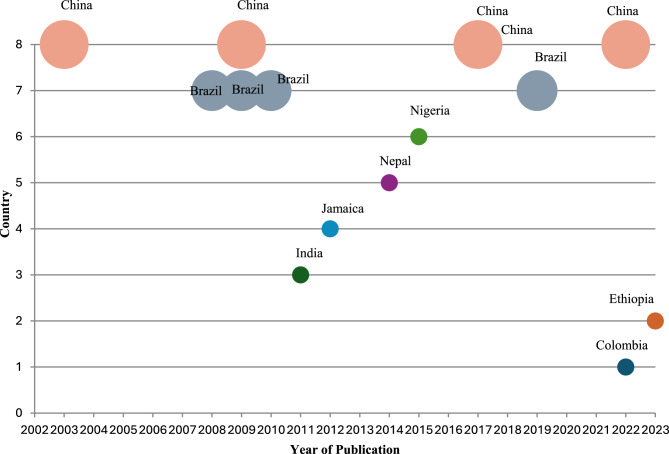
Bubble plot of included articles by publication year and author-affiliated countries of LMICs

In terms of geographical location, these studies were distributed across various regions in LMICs, with five from China [24, 31–33, 36], four from Brazil [26, 28, 30, 35] and one [1] study from each of the following countries Nepal [27], India [13], Jamaica [34], Colombia [25], Nigeria [29] and Ethiopia [15]. The included articles were published between 2003 and 2023 (Fig. 2).

The included studies varied considerably in sample size, ranging from 120 to 184,930 participants. Regarding gender differences among study participants, nearly all studies included both men and women, apart from one study that focussed exclusively on men [31].

Different tools were used to assess AUD and co-occurring mental illness across the included studies. These tools included the Alcohol Use Disorder Identification Test (AUDIT), Alcohol, Smoking and Substance Involvement Screening Test (ASSIST) and the Michigan Alcoholism Screening test (Geriatric Version (MAST-G) to evaluate alcohol abuse and probable dependence [15, 32, 35]. Depressive symptoms were assessed using the Hamilton Depression Rating Scale (HDRS), Centre for Epidemiological Studies Depression Scale (CES-D-10), Zung Self-rating Depression Scale (ZSDS), World Health Organization Composite International Diagnostic Interview (CIDI) version three and Geriatric Depression Scale (GDS) [24, 29, 33].

Cognitive impairment was assessed using Mini Mental State Examination Modified (MMSE-M) and the adapted 10-Word Delay Recall Test (10-WDRT). Functionality was assessed using the Barthel Index for Activities of Daily Living (BADL) and Activities of Daily Living (ADL) scale to measure living ability [13, 25, 29, 32].

The included studies explored a range of key issues related to AUD and co-occurring mental illness among older adults. These studies specifically, reported on the prevalence and patterns of AUD in mental illness, gender differences in drinking patterns, associated risk factors and mental health outcomes.

To provide a more comprehensive picture, one included study reported the religious characteristics and their associations with tobacco, alcohol, and depression among older adults [30]. The following table highlights the main themes across the included studies (Table 3).

**Table 3:** Main themes across the included studies

Themes	Key findings
Prevalence and pattern of alcohol use	• Lifetime alcohol use:10% to 89.3% • Current alcohol use: 15.6% to 52.4% • Heavy drinking in men: 8.2–10% • Moderate alcohol use:42.4%, • Alcohol abuse or probable dependence:26.5% • Alcohol use disorder:27.5% • Decline with alcohol use with increasing age
Associated factors	• Social determinants: low education, low socioeconomic status, rural/urban disparities • Risk increases with isolation and living alone • Cultural norms and religiosity factors
Gender differences in drinking patterns	• Men were more likely to consume alcohol and experience • Women were more susceptible to mental and cognitive health effects at lower alcohol levels • Former and current alcohol use in women associated to loneliness and depression
Mental health outcomes	• Depression and cognitive impairment • Suicidal ideation and poor sleep quality • Functionality impairment • Concurrent substance use problems

### Prevalence and patterns of alcohol use among older adults with mental illness

Across the included studies, the prevalence and patterns of alcohol use among older adults varied considerably across different levels and forms of consumption. Lifetime alcohol use ranged widely, with between 10% and 89.3% [15, 26, 28, 35]. This range includes both lifetime misuse (10%) and former users (19.5%), indicating that a large proportion of older adults had some level of alcohol exposure across their lifespan.

Similarly, current alcohol use was reported in 15.6% to 52.4% of older adults, with roughly one third continuing to drink regularly [27, 35].

The prevalence of AUD was reported at 27.5%, indicating that more than one in four older adults met the criteria for problematic alcohol use [15]. Likewise, heavy alcohol consumption (CAGE score of ≥2), was reported in 8.2% of the total sample, whereas mild to moderate drinking (CAGE score of < 2) accounted for 42,4%, showing that moderate alcohol use was more common [28]. Another study revealed that 26.5% of older adults were identified as having alcohol abuse or probable dependence [35]. Age differences followed a clear declining pattern, with the highest prevalence of alcohol use among those aged 60–69 years (29.4%), followed by 70–79 years (17.6%), 80–89 years (11.8%) and 90 years and older (9.0%), suggesting alcohol use tended to decline with age [34]. Religious factors also influenced drinking behaviours. One large scale study involving 6,962 participants showed that changes in religious affiliation or practice were associated with a 31% higher risk of alcohol abuse or dependence [30].

**Fig. 3:**
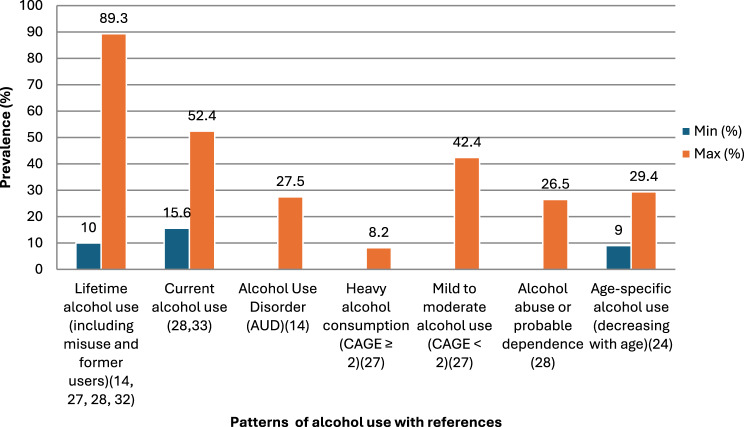
Range bar chart showing different classification of alcohol use prevalence among older adults

Overall, the evidence highlights substantial variability in alcohol use across contexts and subgroups. While lifetime and current use remain relatively high, heavy or dependent use though less common remains clinically significant, particularly among men. Patterns suggest that alcohol use tends to decline with age and social or religious factors may serve as moderating influences on the prevalence of alcohol related problem in older adults (Fig. 3).

### Associated factors of alcohol use and co-occurring metal illness among older adults

Across the reviewed studies, several associated factors were identified as contributing to AUD and co-occurring mental illness among older adults. Being male and at the younger end of older age range consistently associated with higher risk, while concurrent substance use particularly smoking was also associated with increased alcohol related problems [27, 29, 30, 32, 34, 35]. Socioeconomic challenges and psychosocial factors were also associated with higher risk of AUD and mental illness [13, 24, 28, 29, 32, 35] (Table 4).

**Table 4:** Associated factors of aud and co-occurring mental illness among older adults

Category	Specific associated factors	Association	Study references
Demographic factors	Being male, relatively younger age within the older population	Increased risk of AUD and co-occurring mental illness	[27, 29, 30, 32, 34, 35]
Behavioural factors	Concurrent substance use, particularly current smoking	Positively associated with AUD and mental illness	[13, 32, 36]
Socioeconomic and environmental factors	Low educational attainment (especially among women), low socioeconomic status, Living alone, rural or urban residence (depending on the context), disadvantaged community settings and poor housing conditions	Increased risk of alcohol use and mental illness	[24, 28, 29, 32, 35]
Health related and psychosocial factors	Chronic health conditions, sleep problems, suicidal ideation or attempts; being unmarried	Positively associated with AUD and co-occurring mental illness	[13, 24, 29]

### Gender differences in alcohol use and co-occurring mental illness

Across the seven of the included studies, men were generally more likely than women to consume alcohol, reflecting a consistent gender gap in drinking behaviours among older adults [26–30, 32, 34, 36].

There are also several nuanced differences that emerged in the patterns and consequences of alcohol use between men and women. While men tended to report higher rates of alcohol consumption, women appeared more vulnerable to poor mental health outcomes, including depression, loneliness and cognitive impairment [13, 31, 35, 36].

In terms of drinking frequency, a smaller proportion of women reported current alcohol use compared to men, yet women who drank were more likely to engage in frequent drinking and experience poorer mental health [29, 31].

Socioeconomic and educational factors also played a gendered role. Low education levels were associated with heavy drinking among women, while low socioeconomic status was a common factor associated with heavy drinking in both men and women. In contrast, mild to moderate alcohol use (CAGE score < 2) was more common among older adults with higher education and socioeconomic status compared to those who abstained from alcohol. This trend held true for both genders [35].

**Fig. 4:**
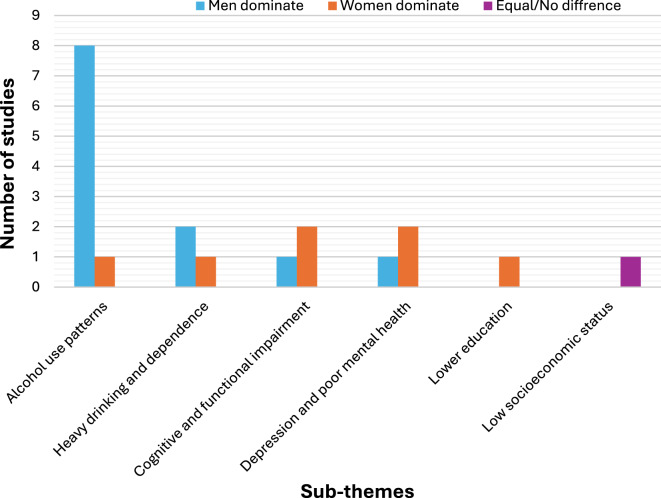
A grouped bar chart showing gender differences in alcohol use and co-occurring mental illness

Collectively, these findings highlight distinct gender differences in alcohol consumption patterns and associated outcomes among older adults, suggesting that women were more susceptible to the psychosocial and cognitive impacts of alcohol use despite lower overall consumption levels (Fig. 4).

### Mental health outcomes associated with alcohol use among older adults

Across the included studies, depression and cognitive impairment are the most reported mental illnesses co-occurring with AUD in older adults. Specifically, seven studies identified depression [25, 26, 30, 34–36] as a frequent comorbidity, while another seven studies reported cognitive impairment ranging from mild decline to dementia [13, 15, 27–29, 32, 33]. One study did not specify a particular type of mental illness but provided data on gender differences in general mental health, smoking, drinking and chronic diseases among older adults [31].

Finding on the relationship between alcohol use and depression were mixed. Several studies showed that excessive or daily drinking significantly increased with the risk of depression, whereas one study reported that older men had a 47% lower risk of depressive symptoms compared to women [24].

Additional factors influenced these relationships; older adults who smoked were 56% more likely to experience cognitive impairment and twice as likely to report alcohol use [13, 26]. Socio-cultural factors such as lack of religious affiliation or change in religious practice also increased the likelihood of alcohol use [30].

Overall, the review suggests that depression and cognitive impairment are the most prevalent co-occurring mental illness in older adults with AUD, with the risks increasing under heavy, frequent alcohol consumption (Table 5).

**Table 5:** Mental health outcomes associated with alcohol use among older adults

Mental health outcomes	Associated alcohol use patterns	Key findings	Study references
Depression	Moderate to heavy drinking, current and lifetime alcohol use	Several studies reported a higher risk of depression with drinking, while one found lower depression symptoms rate with drinkers	[25, 26, 30, 34–36]
Cognitive impairment/dementia	Daily/excessive drinking, current and lifetime use of alcohol	Daily drinking/current was associated with a higher likelihood of cognitive impairment, while overall alcohol use also showed an increased risk	[13, 15, 27–29, 32, 33]
Poor sleep quality and suicidal ideation	Alcohol use disorder	AUD associated to poor sleep and suicidal thoughts	[15]
Functional impairment	Lifetime alcohol misuse	Lifetime alcohol use doubled the functional impairment	[26]
Concurrent substance use Problems	Smoking and alcohol use combined	Co-users had 56% higher cognitive impairment likelihood	[13, 26]

Detailed characteristics of the included studies and a summary of key findings are presented in (Table 6).

**Table 6:** The characteristics of included studies and summarised key results

S.No	Author(s) and year of publication	Aims/purpose	Country	Sample size	Study type/source	Types of mental illness co-occurring with AUD	Factors associated with AUD and co-occurring mental illness	Key findings that relate to the review question/s
1	Yujun Liu et al. [24]	To assess the relationship between alcohol consumption and depressive symptoms among older adults	China	7,601	Community based cross sectional study	Depression	1. Being female 2. Living alone (vs. living with a spouse only) 3. Living in a rural community (vs. urban) 4. Living in a community with lower socioeconomic status 5. Living in poor housing conditions 6. Having poorer self-reported physical health and more limitations in activities of daily living and instrumental activities of daily living	• Most older adults in China (70%) are not current drinkers, and heavy drinking is much more common among older men (92.91%) compared to older women (7.09%). • Being male decreases the risk of experiencing depressive symptoms by 47%, living in a rural area increases the risk by 39%, and living in a higher SES community decreases the risk by 10%. • The authors conclude that the findings provide new insights into alcohol consumption and depressive symptoms among older adults in China and highlight the need for culturally sensitive alcohol prevention programs targeting this population.
2	Yen-Chang Chang et al. (2022) [36]	Assessing the associations between smoking and alcohol consumption with loneliness, depression, and loss of interest among Chinese older adults.	China	5,874	Community based longitudinal study design	Depression	1. Former alcohol users had higher odds of reporting depression (adjusted odds ratio [AOR] = 1.39, 95% CI: 1.01, 1.91; *p* < 0.05), while current alcohol users did not. Current and former smoking statuses were positively associated with depression (all *p* < 0.05). However, only current smoking status was positively associated with loss of interest (*p* < 0.05). 2. Among older women, former alcohol consumption was positively associated with loneliness, and current alcohol consumption was positively associated with depression.	• The prevalence of alcohol use status was 50.1% non-users, 19.5% former users, and 30.4% current users. • The authors conclude that among older Chinese men, current smoking is associated with higher odds of depression and loss of interest, while among older Chinese women, current alcohol consumption is associated with higher odds of depression. • The authors suggest several policy implications, such as promoting healthy hobbies, incorporating mental health into alcohol cessation programs, and adjusting cultural norms around smoking and drinking.
3	Roger C et al. (2012) [34]	To assess the prevalence of alcohol use among older Jamaicans and to explore the relationships between alcohol use and age, sex, depressive symptoms, and life satisfaction	Jamaica	2,943	Community based cross sectional study	Depression	Current alcohol use among older Jamaicans was associated with being male, younger age, lower levels of depressive symptoms, and both high and low levels of life satisfaction.	• Current alcohol use was reported by 21.4% of older Jamaicans but declined with increasing age. • Alcohol use was much more prevalent among men (37.6%) compared to women (6.2%). • Current alcohol use was associated with lower levels of depressive symptoms and was more common among those with either very high or very low life satisfaction. • Authors recommended that future research which should incorporate the quantification of past and current alcohol use, as well as participants’ reasons for drinking, to better understand the relationships between alcohol use, depression, and life satisfaction. And include additional measures of life satisfaction to strengthen the analyses
4	N. Castellanos-Perilla et al. [25]	To explore how different depressive symptoms might contribute to different patterns of alcohol consumption in Colombian older adults living in the community, in order to inform interventions that target specific depressive symptoms associated with particular drinking patterns.	Colombia	19,004	Community based secondary analysis of a cross-sectional study	Depression	Older adults with dysphoric depressive symptoms like feeling worthless, hopeless, and having a low mood tend to have lower weekly alcohol consumption frequency. Older adults with withdrawal/apathy related depressive symptoms like dropping activities and preferring to stay home tend to have higher weekly alcohol consumption frequency. Older adults with lack of energy are more likely to be moderate drinkers and less likely to be heavy drinkers.	• Lower weekly drinking frequency and higher number of drinks per serving are associated with higher total GDS (depression) scores. • The prevalence of moderate drinking is 1.47% and heavy drinking is 10% in the sample. • The”feeling without energy” symptom is associated with a higher likelihood of moderate drinking (OR 1.94, 95% CI 1.39–2.69) but lower likelihood of heavy drinking (OR 0.55, 95% CI 0.40–0.70). • The total GDS (depression) score is associated with a higher number of drinks per serving (incidence rate ratio 1.01, 95% CI 1.01–1.02). More studies are needed to replicate and improve methods with more detailed surveys regarding alcohol use and longitudinal designs and to establish directionality in the association between the onset of depressive symptoms and changes in drinking habits.
5	Sergio Lu´ıs Blay et al. [26]	To examine the sociodemographic correlates and health effects associated with lifetime alcohol misuse in community-dwelling elderly people in Brazil	Brazil	6961	Community based secondary analysis of a cross-sectional study	1.Psychiatric disorders in general, with a doubling of the odds for those endorsing 2+ alcohol misuse items 2. Specifically, depression was the most prevalent psychiatric disorder associated with alcohol misuse	1. Men were 8 times more likely than women to endorse one alcohol misuse item (OR = 8.19, 95% CI = 5.56–12.06). 2. As age increased (60–69,70–79,80+), endorsement of two or more alcohol misuse items decreased. (OR = 0.69, 95% CI = 0.49–0.96), OR = 0.42, 95% CI = 0.22–0.79) (OR = 1.42, 95%CI = 1.03–1.95) respectively. 3. Persons with lower income and non-white, particularly African Brazilian, race/ethnicity were nearly 3 times more likely to endorse two or more alcohol misuse items. (OR = 2.85, 95%CI = 1.78–4.57). 4. Tobacco users were twice as likely as non-users to report alcohol misuse. (OR = 2.02, 95%CI = 1.43–2.83) 5. Endorsing two or more alcohol misuse items doubled the odds of multiple ADL problems and psychiatric disorders (OR = 2.19, 95% CI = 1.58–3.03).	• 10.6% of the sample reported lifetime alcohol misuse, with 25.3% of men and 2.9% of women endorsing at least one item. • Lifetime alcohol misuse was more common in certain sociodemographic groups, including men, younger elderly (aged 60–69), those with lower income, and non-white (especially African Brazilian) individuals. • Endorsing more than one item indicating lifetime alcohol misuse was associated with poorer functional status, respiratory problems, and psychiatric disorders, but was protective against vascular conditions. • Authors suggest that future research should examine the causal relationship between alcohol misuse and health outcomes.
6	Prakash Thapa et al., [27]	To find out the prevalence of different psychiatric morbidities in elderly population and to find out if there are any age and gender specific differences.	Nepal	120	Facility based cross-sectional study	Depression and Dementia	1.Alcohol dependence syndrome and dementia were more common in male elderly patients compared to females. 2. The prevalence of alcohol-related disorders decreased with increasing age	• Depressive disorder was the most common psychiatric diagnosis, affecting 26.7% of the elderly patients. • The risk of dementia increased significantly with age, with those over 75 having much higher odds of dementia compared to those 75 and under. • Alcohol dependence syndrome and dementia were both significantly more common in male patients compared to female patients • Authors conccluede that prospective study with structured assessments is recommended to address and the development of age-specific diagnostic criteria may be needed in the future.
7	Marcos A. Lopes et al., [28]	To estimate the prevalence of alcohol related problems in an elderly population from Brazil and to investigate their association with cognitive and functional impairment (CFI) and dementia.	Brazil	1,145	Community based cross-sectional study	Dementia	1.Sociodemographic factors: Being male, low education (in females), and low socioeconomic status are associated with heavy alcohol use. 2. Heavy alcohol use is associated with higher rates of cognitive impairment and dementia, particularly in females. 3. Mild-moderate alcohol use may be associated with lower rates of cognitive impairment compared to no alcohol use, but this relationship is not as strong.	• 1. The prevalence of heavy alcohol use (CAGE ≥ 2) was 8.2% in the total sample, with a much higher rate in males (17.4%) compared to females (3.0%). 2. Heavy alcohol use was associated with lower education only in females, and with lower socioeconomic status in both genders. 3. Mild-moderate alcohol use (CAGE < 2) was associated with higher education and socioeconomic status compared to no alcohol use for both genders. 4. Heavy alcohol use was associated with higher rates of cognitive and functional impairment (CFI) and dementia compared to mild-moderate alcohol use, particularly in females. • The authors state that there is still insufficient evidence to recommend mild-moderate alcohol use as a protective factor against cognitive disorders in the elderly, due to methodological limitations in their study and the need for more research in this area.
8	Mariana Silva et al., (2019) [35]	To assess the prevalence and the factors associated with alcohol abuse and probable dependence among older individuals.	Brazil	614	Community based cross-sectional study	Depression	1. Being male was associated with a 6.67-fold greater risk of alcohol abuse and probable dependence (*p* = 0.001). 2.The presence of depressive symptoms was associated with a 2.74-fold greater risk of alcohol abuse and probable dependence (*p* = 0.023)	• The prevalence of abuse and probable alcohol dependence among older adults was 26.5%. • Being male was associated with a 6-fold greater risk of alcohol abuse and dependence. • The presence of depressive symptoms was associated with a 2.74-fold greater risk of alcohol abuse and dependence • The authors recommend using these findings to inform policy decisions related to aging and mental health, as well as to improve training and practice of nursing professionals in caring for elderly with alcohol abuse and dependence.
9	V O Lasebikan et al., [29]	To determine the prevalence of lifetime and 7-day alcohol consumption, as well as 7-day excessive drinking, and to examine the association between 7-day excessive drinking and various chronic health conditions in the elderly population	Nigeria	2,152	Community based cross-sectional study	Cognitive impairment	1.Relatively younger age 2. urban residence 3. chronic health conditions 4. unmarried	• 54.8% of older adults were lifetime abstainers from alcohol • 12.9% of elderly men and 3.8% of elderly women reported alcohol use in the past 7 days • Excessive alcohol consumption ( > 1 unit/day) was only found in 3.3% of men • Excessive alcohol consumption in the past 7 days was associated with chronic back/neck pain and cognitive impairment • The authors conclude that a relatively high proportion of elderly Nigerians consume alcohol, and that excessive drinking was associated with chronic pain and cognitive impairment which could inform public health policies and interventions targeting alcohol misuse in the elderly in Nigeria.
10	Sergio Lu´ıs Blay et al. [30]	To assess the religious characteristics of older subjects and the associations of these characteristics to the use of tobacco, alcohol, and depression.	Brazil	6,961	Community based cross-sectional study	Depression	Religious affiliation, religious change, orienting-motivating force, and social religiosity, which were found to be independently associated with alcohol abuse/dependence, and depression	• Not having a religious affiliation was associated with an 88% increased risk of alcohol abuse/dependence • Experiencing a religious change was associated with a 31% increased risk of alcohol abuse/dependence • Considering religion as an orienting-motivating force was associated with a 38% increased risk of depression • Participating in social religious activities was associated with a 16% reduced risk of depression Authors concclude that the study indicates that religiosity may be a useful factor to consider in developing guidelines and interventions to facilitate healthful behaviours in the older adults.
11	Shibin Wang et al., [31]	To investigate the gender differences in general mental health, smoking, drinking, and chronic diseases among older adults	China	4,115	Community based cross-sectional study	Not specified in this study	Frequent drinking was gender, with men being more likely to be frequent drinkers compared to women.	• Women frequent drinkers (OR 0.11 95% CI = 0.05 to 0.21) *p* < 0.001 had higher prevalence (from *n* = 2198 the prevalence was 28.9%) of poor mental health compared to men (OR 1.59 95% CI = 1.31 to 1.92) *p* < 0.001. • Older women were less likely to be current smokers and frequent drinkers but had a higher prevalence of poor mental health compared to older men • The prevalence rate of chronic diseases and multi-morbidities were higher in women (from *n* = 2198 the prevalence was 92.9%) than in men (*n* = 1917 the prevalence was 86.6%). (OR 1.83, 95% CI = 1.36–2.45) *p* < 0.001, (OR 1.89, 95% CI = 1.57–2.28) *p* < 0.001respectively • The authors conclude that health professionals and policymakers should pay special attention to the higher rates of smoking and drinking in older men. They recommend that more epidemiological research as well as large-scale studies focusing on gender differences in health conditions among older Chinese populations.
12	Wolde et al., [15]	To assess the prevalence and associated factors of alcohol use disorder among the elderly individuals	Ethiopia	422	Community based cross-sectional study	Cognitive impairment, poor sleep quality and suicidal ideation	Factors independently associated with AUD in the older adults were: - Cognitive impairment (AOR = 2.53, 95% CI = 1.18–5.42) - Poor sleep quality (AOR = 2.67, 95% CI = 1.10–6.44) - Physical illness (AOR = 3.27, 95% CI = 1.49–7.15) - Suicidal ideation or attempt (AOR = 2.07, 95% CI = 1.06–4.40)	• The prevalence of alcohol use disorder, current alcohol use, and lifetime alcohol use among older adults was 27.5%, 52.4%, and 89.3%, respectively. • Alcohol use disorder was associated with cognitive impairment, poor sleep quality, chronic medical illness, and suicidal ideation. • Screening for alcohol use disorder and its associated risk factors, and providing appropriate management, is crucial to prevent further complications in the elderly population. • Authors recommended that screening and intervention for AUD and its associated factors is crucial, and that proper health education about alcohol misuse and related factors should be provided to the older adult population. They also recommend that further research is needed to determine if their findings apply to other elderly populations in Ethiopia and other regions.
13	Zhou Huadong et al., [32]	To assess the relationship between cigarette smoking, alcohol drinking, and cognitive impairment among elderly people	China	3,012	Community based cross-sectional study	Cognitive impairment	1. Cigarette smoking, with current smokers having a significantly higher risk of cognitive impairment compared to those who have never smoked. 2. Alcohol drinking, with those who drink alcohol every day having a significantly higher risk of cognitive impairment compared to occasional drinkers. 3. Other factors such as age, sex, occupation, and education level were also associated with cognitive impairment.	• The prevalence of cognitive impairment was 3.4% in abstainers, 6.4% in those who drank weekly, and 17.5% in those who drank daily • Drinking alcohol every day was associated with a 3.47 times higher risk of cognitive impairment compared to occasional drinking (95% CI: 1.79–6.71) • Past smoking was not significantly associated with cognitive impairment risk. • The authors conclude that cessation of smoking and reduction of drinking could be considered as part of a strategy to reduce the incidence of cognitive impairment.
14	Gelin Xu et al., [33]	To examine the relationship between alcohol consumption and the risk of developing dementia in a cohort of older patients with mild cognitive impairment	China	176	Prospective cohort study	Cognitive impairment	1. Heavy alcohol consumption is associated with negative effects on cognitive function and increased risk of dementia. 2. Both heavy and abstinent alcohol consumption are associated with increased risk of cognitive decline, while light-moderate consumption may be protective. 3. There is a J-shaped relationship between alcohol consumption and dementia risk, where light-moderate drinking is associated with the lowest risk.	• Light-moderate alcohol drinkers had better cognitive function (as measured by MMSE) compared to abstainers (*p* < 0.05) and heavy drinkers (*p* < 0.01) 2 years after MCI diagnosis. • Patients who consumed up to 300 kg of alcohol prior to MCI diagnosis had less cognitive decline compared to those who consumed no alcohol (*p* < 0.05) or over 300 kg (*p* < 0.01). • Heavy drinkers had a higher risk of developing dementia compared to abstainers (*p* < 0.05) and light-moderate (*p* < 0.05) drinkers 2 years after MCI diagnosis. • Authors concclude tha further research is needed to understand the mechanisms behind this relationship.
15	T Muhammad et al., (2011) [13]	To explore the factors associated with cognitive impairment, especially the role of alcohol consumption, smoking, and chewing tobacco, and to determine if there is an interaction effect between tobacco use (smoking and chewing) and alcohol consumption on cognitive impairment among older adults	India	9,453	Community based cross-sectional study	Cognitive impairment	1.Smoking tobacco, consuming alcohol, and the combination of smoking tobacco and consuming alcohol. 2. The analysis also controlled for several potential confounding variables, including socioeconomic and demographic factors, health status, and functional ability.	• Older adults who smoke tobacco have a 24% higher likelihood of cognitive impairment compared to those who do not smoke. • Older adults who consume alcohol have a 30% higher likelihood of cognitive impairment compared to those who do not drink. • Older adults who both smoke and consume alcohol have a 56% higher likelihood of cognitive impairment compared to those who do neither. • Authors concclude that implementing preventive measures to counter the risk factors for cognitive impairment, including alcohol drinking, smoking, and chewing tobacco

### Critical appraisal of studies

The studies included in this study were all descriptive observational studies. Their quality was assessed by three researchers using the CASP checklist, which is designed for evaluating systematic reviews and meta-analyses of observational studies [23]. From three longitudinal studies at least two studies met (82%) of the quality criteria and one study met (72%) quality criteria [27, 33, 36]. Of the twelve cross-sectional studies, five studies met (100%) quality criteria [13, 15, 25, 29, 30] and the remaining seven studies met at least (75%) quality items [24, 26, 28, 31, 32, 34, 35]. The quality assessment results of each study are found in (Tables 7 and 8).

**Table 7:** Risk of bias assessment for cross-sectional studies

Studies	Were the criteria for inclusion in the sample clearly defined?	Were the study subjects and the setting described in detail?	Was the exposure measured in a valid and reliable way?	Were objective, standard criteria used for measurement of the condition?	Were confounding factors identified?	Were strategies to deal with confounding factors stated?	Were the outcomes measured in a valid and reliable way?	Was appropriate statistical analysis used?
Yujun Liu et al. [24]	Yes	Yes	No	Yes	Yes	Yes	No	Yes
Roger C et al. (2012) [34]	Yes	Yes	Yes	Yes	No	No	Yes	Yes
N. Castellanos-Perilla et al. [25]	Yes	Yes	Yes	Yes	Yes	Yes	Yes	Yes
Sergio Lu´ıs Blay et al. [26]	Yes	Yes	No	Yes	Yes	Yes	No	Yes
Marcos A. Lopes et al., [28]	Yes	Yes	Yes	Yes	Yes	Yes	No	Yes
Mariana Silva et al.,(2019) [35]	Yes	Yes	Yes	Yes	Yes	No	Yes	Yes
V O Lasebikan et al., [29]	Yes	Yes	Yes	Yes	Yes	Yes	Yes	Yes
Sergio Lu´ıs Blay et al. [30]	Yes	Yes	Yes	Yes	Yes	Yes	Yes	Yes
Shibin Wang et al., [31]	Yes	Yes	No	Yes	Yes	Yes	No	Yes
Wolde et al., [15]	Yes	Yes	Yes	Yes	Yes	Yes	Yes	Yes
Zhou Huadong et al., [32]	Yes	Yes	No	Yes	Yes	Yes	Yes	Yes
T Muhammad et al., (2011) [13]	Yes	Yes	Yes	Yes	Yes	Yes	Yes	Yes

**Table 8:** Risk of bias assessment for cohort studies

Studies	Were the two groups similar and recruited from the same population?	Were the exposures measured similarly to assign people to both exposed and unexposed groups?	Was the exposure measured in a valid and reliable way?	Were confounding factors identified?	Were strategies to deal with confounding factors stated?	Were the groups/participants free of the outcome at the start of the study (or at the moment of exposure)?	Were the outcomes measured in a valid and reliable way?	Was the follow up time reported and sufficient to be long enough for outcomes to occur?	Was follow up complete, and if not, were the reasons to loss to follow up described and explored?	Were strategies to address incomplete follow up utilized?	Was appropriate statistical analysis used?
Yen-Chang Chang et al. (2022) [36]	Yes	Yes	Yes	Yes	Yes	No	No	Yes	Yes	Unclear	Yes
Prakash Thapa et al., [27]	Yes	Yes	Yes	Yes	Yes	No	No	Yes	Yes	Yes	Yes
Gelin Xu et al., [33]	Yes	Yes	Yes	Yes	Yes	Yes	Yes	No	Yes	No	Yes

The quality assessment results of each included cross-sectional study have been thoroughly evaluated and presented in (Table 7).

The quality assessment results of each included cohort study have been thoroughly evaluated and provided in (Table 8).

## Discussion

### Overview

This review aimed to synthesize existing literature on AUD and co-occurring mental illness among older adults in LMICs, focusing on identifying prevalence and pattern trends, associated risk factors and mental health outcomes/psychosocial impacts. It revealed substantial variation across studies in AUD and mental health outcomes as well as in measurement tools and diagnostic criteria. Alcohol related outcomes varied widely across studies, such as lifetime alcohol use, current use, heavy drinking, alcohol abuse or dependence studied with different mental health outcomes. Because of this heterogeneity, conducting a meta-analysis was not feasible. Therefore, a narrative review with systematic search was most appropriate approach to integrate and interpret diverse findings while maintain methodological rigor.

The findings reveal several consistent themes across studies, including prevalence and patterns, gender differences in drinking patterns, alcohol related mental health outcomes and associated factors.

These findings highlight the need for age appropriate, culturally sensitive interventions and policies to address the dual diagnosis of AUD and mental illness in older adults. Although the reviewed studies consistently identified the associations between alcohol use and mental health outcomes, these patterns should be viewed as indicative rather than casual, reflecting the predominance of cross-sectional study designs that limit the ability to determine directionality within these relationships.

### Prevalence and patterns of alcohol use among older adults with mental illness

Across studies, alcohol use among older adults showed wide variation. Lifetime use ranged from 10% to 89.3% [15, 26, 28, 35], while current use was reported in 15% to 52.4% of participants [27, 35]. About one in four older adults (27.5%) met the criteria for Alcohol Use Disorder (AUD) [15].

The possible reason for this could be older adults may have initiated drinking earlier in life when alcohol was more socially acceptable or available in their cultural and social contexts, thus contributing to high lifetime prevalence. The difference in current alcohol use might be influenced by other chronic health issues of older adults, long term medication follow up and variation of social roles in later life. These factors may contribute to some individuals in this demography to reduce or stop drinking while others proceed. Furthermore, variation in measurement of AUD, study designs, cultural norms and sample size and recall accuracy in older adults could account for discrepancies across studies.

Alcohol use tended to decline with age, being relatively highest among those aged 60 and 69 years (29.4%) followed by individuals between 70 and 79 years (17.6%), then by individuals between 80 and 89 years (11.8%) and then by individuals of 90 years of age or older (9.0%). In one of the included studies [34] this could be explained by age related health concerns, other medication use, decreased social drinking opportunities and lifestyle changes. This suggests that despite the research indicating a decline in alcohol consumption as individual ages, a substantial cohort of older adults continue to consume problematic quantities of alcohol.

### Sociodemographic factors and religious influences

Sociodemographic factors such as low educational attainment particularly among females, lower socioeconomic status, living alone and poor physical health were significantly associated with increased alcohol use [24, 28, 32]. These finding aligns with study from developed nations, which have shown that psychosocial factors like social isolation and loneliness, limited awareness, financial stress and cultural factors can influence AUD and co-occurring mental illness [37]. This could be explained by limited awareness of the health risks associated with alcohol use, along with social normalization and easy availability of locally produced alcoholic beverages may further contribute to harmful alcohol use, particularly in LMICs. Older adults living alone or experiencing chronic illness may also use alcohol to cope with loneliness or physical discomfort. These findings highlight the urgent need for targeted alcohol education and awareness programs at both community and institutional levels.

Religious affiliation and practice may also influence drinking behaviours. Evidence from one study indicated that shift in religious practice was associated a 31% increased risk of alcohol use [30]. This may reflect the potential psychosocial and behavioural roles that religion can play. Changes in religious engagement might be accompanied by psychological distress, social withdrawal or reduced behavioural constraints which, may in turn contribute to higher alcohol use.

### Gender differences in alcohol use and mental health outcomes

Across several studies, older men were consistently found to engage in alcohol use at higher rates than women, showing heavy drinking was more common in men than women, with rates between (8.2%) and (17.4%) in men versus (3%) to (7.09%) in women. However, its association with cognitive, functional impairment and dementia appeared stronger in women.

Mental health outcomes also varied by gender, as reported women frequent drinkers showed significantly higher rates of poor mental health than men and being male was associated with a lower risk of depressive symptoms in one study. Overall, the former men drinkers had increased odds of depression. Additionally, socioeconomic factors influenced alcohol use patterns, with lower education associated with heavy drinking in women and lower socioeconomic status associated with heavy drinking in both genders [13, 26–29, 31, 32, 34–36].

These findings emphasize the importance of gender sensitive health promotion strategies, addressing higher drinking rates in older men and the heightened mental health risks faced by older women. This finding is consistent with studies conducted in developed nations, where research on AUD and co-occurring mental illness showed that males typically having higher rates of alcohol and illicit drug use than females [38].

However, in most studies the sample size for older women was much smaller than for older men, particularly for smoking and alcohol use, which may have limited the statistical power and generalizability of the findings for older women. Another possible reason for this condition relates to long standing gender norms, coping mechanisms and health seeking behaviours. Historically, older men may be more socially conditioned to engage in alcohol use as an accepted or normalized means of stress relief or social bonding, while women may face greater stigma for drinking, contributing to lower reported rates [36].

However, when women do engage in heavy drinking, biological differences such as lower body water content and differences in alcohol metabolism may increase their vulnerability to alcohol related health consequences, including cognitive decline and functional impairment [39]. Furthermore, older women had a greater chance of experiencing social isolation, widowhood and caregiving burdens all of which can contribute to poor mental health outcomes. This may reflect why mental health challenges like depression and loneliness are more prevalent among older women, especially those who consume alcohol. Older men reported lower depressive symptoms but higher physical health risks due to heavier alcohol use than women. This gender specific pattern may reflect cultural and social contexts rather than protective biological effects. In many contexts, men drink for social or leisure purposes, while women face greater stigma and isolation to drinking [24]. Such differences may explain why moderate alcohol use appears linked to lower depressive symptoms among men but not among women.

This highlights the need for health professionals and policymakers to pay special attention to the higher prevalence of poor mental health in older women and higher drinking rates in older men. It also provides important epidemiological insight into modifiable risk factors that can guide targeted health promotion and disease prevention strategies for older adults [31].

### Mental health outcomes

Across the included studies, depression and cognitive impairment were the most reported mental illness co-occurring with AUD in older adults. Other associated conditions included poor sleep quality and suicidal ideation [15, 24–36]. This finding is consistent with several studies from developed nations [40, 41]. This could be explained as these comorbidities can have a bidirectional relationship, mental illness may lead individuals to use alcohol as self-medication, while alcohol use can trigger or worsen mental illness [8, 9]. Such conditions are frequently underrecognized [10], yet they impose significant psychological, social, and economic burden on the older adults, their families and the community [11]. Early detection and integrated intervention can improve outcomes [12].

Two studies in LMICs reported that concurrent substance use particularly tobacco use was significantly associated with AUD and co-occurring mental illness [13, 26]. This could be explained by the pattern among older adults with other substance use problems to concurrently use multiple substances, where one substance often acts as a gateway to others [42]. These findings underscore the complex and multifaceted nature of co-occurring mental illness in this population, highlighting the need for comprehensive and integrated approaches to assessment, treatment and support.

### Implications for practice

The findings of this review have important implications for healthcare practice, particularly in LMICs, where health systems often operate with limited resources and insufficient geriatric mental health services [43, 44]. Older adults with AUD and co-occurring mental illness present unique clinical, psychosocial and service-related challenges that differ substantially from those of younger individuals [45]. Their complex presentations reflected in this review as depression, cognitive impairment, poor sleep and suicidal behaviours demand integrated and age sensitive approaches to assessment and management [24–26, 30, 34–36].

In contrast to high-income countries, where integrated behavioural services for older adults are increasingly established [46, 47], LMICs often lack the research, service frameworks and most primary health care providers lack sufficient training and formal education in geriatric psychiatry and AUD [43, 44, 48, 49]. Strengthening care in these settings requires workforce development through geriatric psychiatry and dual diagnosis training, embedding screening and management of AUD and dual diagnosis within primary care and promoting multidisciplinary collaboration.

We hope this review will guide clinicians, researchers and policy makers in translating these findings into practical strategies that promote equitable and age-appropriate care. Clinicians can enhance routine screening and ensure continuity of care. Researchers can further investigate the causal pathways involving alcohol use to specific mental health outcomes and generate locally driven evidence on effective intervention. Policymakers can prioritize this population within national health strategies. Together, these efforts can help integrated and sustainable care systems for older adults with AUD and mental illness in LMICs.

### Gaps and limitation of the review process

This review followed rigours procedures, including protocol registration on PROSPERO, reporting with the references to the PRISMA 2020 guidelines where applicable and use of the CASP to assess the study quality. Despite these measures, several limitations remain. The included studies were highly heterogeneous across definitions and outcome assessments, limiting potential for quantitative synthesis. As a result, a narrative synthesis was undertaken which, while appropriate for such variability, carries an inherent risk of interpretative bias and limited reproducibility compared to statistical meta-analysis. The reporting was informed by the PRISMA guidelines where applicable and SWiM approach, were applied to enhance transparency and consistency in reporting.

The review included only studies published in English, which may have excluded relevant evidence from non-English speaking LMICs, introducing potential language and regional bias. Some of the studies also relied on self-reported data on alcohol use [28, 31, 33, 36], which may be prone to underreporting due to stigma or limited awareness among older adults. Moreover, the predominancy of cross-sectional designs (12 of 15 studies) restricts casual interpretation, while most studies came from China and Brazil, limiting geographic diversity and generalizability to other LMICs regions, especially Africa and Southeast Asia.

Furthermore, due to the nature of comprehensive systematic searches, a small number of records from high-income countries may still appear despite using LMICs filter, as database classifications are periodically updated and some upper-middle-income countries are interchangeable across systems, occasionally shifting into higher income groups and vice versa. However, these records were identified and excluded during title, abstract and full text screening process.

Existing research on dual diagnoses among older adults in LMICs reveals several limitations that impede a comprehensive understanding of this complex issues. Most of the research excluded from this review has been conducted with younger populations and typical individuals under the age of 60, leaving limited understanding of specific care needs, lived experiences and support systems required for older adults with AUD and mental illness.

Overall, these limitations highlights the need for more geographically diverse, longitudinal and methodological robust studies using standardized and validated measures to better understand dual diagnosis among older adults in low-and middle-income countries.

Therefore, future studies should aim to generate new evidence on dual diagnosis and associated factors among older adults to guide the development of culturally appropriate prevention and intervention programs.

## Conclusion

This review shows that AUD and co-occurring mental illness are significant yet underexplored public health issues among older adults in LMICs. Reviews suggests clear gender differences in drinking patterns with men more likely to engage in heavy alcohol use, while women experience higher rates of depression and cognitive impairment related to drinking. Alcohol consumption among older adults was also associated with depressive symptoms, cognitive impairments, suicidal behaviours and poor sleep quality.

A range of associated factors including smoking, low education, low socioeconomic status, chronic illness and social isolation were associated with alcohol related mental health outcomes. Cultural and religious related factors further influenced drinking behaviours and coping patterns in later life. However, much of the evidence is drawn from cross-sectional, self-reported studies concentrated in a few countries, limiting causal interpretation and generalizability across LMICs.

Overall, this review provides a descriptive synthesis of current evidence and underscore the need for more rigorous, longitudinal and culturally grounded research to better understand the dynamics of AUD and co-occurring mental illness among older adults and to inform the design of appropriate and contextually relevant information.

## Data Availability

All the data and materials in this study are available from the corresponding author up on a reasonable request.

## Abbreviations

AUD: Alcohol Use Disorder
AUDIT: Alcohol Use Disorders Identification Test
ASSIST: Alcohol, Smoking and Substance Involvement Screening Test
CAGE: Cut, Annoyed, Guilty, Eye opener
CASP: Critical Appraisal Skills Programme
DSM: Diagnostic and Statistical Manual of Mental Disorders
GHE: Global Health Estimates
GDS: Geriatric Depression Scale
LMICs: Low-and Middle-Income Countries
NESARC: National Epidemiological Survey on Alcohol and Related Conditions
NSDUH: National Survey on Drug Use and Health
PRISMA: Preferred Reporting Items for Systematic reviews and Meta-Analyses
RCTs: Randomized controlled trials
STAR: Supportive Treatment for Addiction Recovery
SUD: Substance Use Disorders
SWiM: Synthesis Without Meta-analysis
WHO: World Health Organization
